# The effect of the use of cassava tuber (*Manihot esculenta*) and Indigofera zollingeriana leaf flour combination as a source of energy supplemented with citric acid in ration on broiler small intestine characteristics and productivity

**DOI:** 10.5455/javar.2022.i616

**Published:** 2022-09-30

**Authors:** Rizki Palupi, Fitri Nova Liya Lubis, Anggriawan Naidilah Tetra Pratama

**Affiliations:** Department of Animal Science, Technology and Industry Study Program Animal Science, Faculty of Agriculture, Sriwijaya University, Ogan Ilir, Indonesia

**Keywords:** Broiler, carcass, performance, *Indigofera zollingeriana* flour, cassava tuber flour

## Abstract

**Objective:**

The study aimed to determine the effect of using a combination of cassava tuber (*Manihot esculenta*) and Indigofera zollingeriana leaf flour as an energy source supplemented with citric acid in the ration on performance, carcass quality, digesta pH, viscosity, and the number of villi in the intestines of broilers.

**Materials and Methods:**

The research design was completely randomized with five treatments and five replications, each containing four broilers. The treatment was a substitution of corn in the ration with a combination of cassava tuber and I. zollingeriana leaf (CIF): without CIF, 5% CIF substitute for corn + 0.2% citric acid, 10% CIF substitute for corn + 0.2% citric acid, 15% CIF corn substitute + 0.2% citric acid, and CIF corn substitute + 0.2% citric acid. Each treatment ration was supplemented with 0.2% citric acid. The variables that were looked at were ration consumption, body weight gain, feed conversion, live weight, percentage of the carcass, percentage of abdominal fat, pH, viscosity, and the number of broiler villi.

**Results:**

This study showed that the combination of CIF flour supplemented with 0.2% citric acid had a significant effect (p < 0.05) on body weight gain, ration conversion, live weight, carcass percentage, and the number of villi in broiler intestines. But it did not have a significant effect (*p* > 0.05) on the amount of feed eaten, the amount of fat in the abdomen, the pH of the digesta, or the thickness of the broilers’ blood.

**Conclusions:**

The combination of CIF flour at a level of 10% supplemented with 0.2% citric acid can be used as an energy source to replace corn without having a bad effect on production performance, carcass quality, and small intestine characteristics of broilers.

## Introduction

Corn is the primary energy source in broiler rations, but its use as broiler feed material competes with the needs of people who consume corn for energy. This will affect corn accessibility and may make corn costs rise, requiring the utilization of elective feed to supplant corn as an energy source in proportion [1,2]. One of the feed ingredients that can be used as an energy source to replace corn is cassava tuber because cassava tuber contains relatively the same energy source as corn. According to Wahyudi and Sudrajat [3], the metabolic energy content of corn is 3,350 kcal/kg, whereas cassava tubers have metabolic energy of 3,519 kcal/kg [4]. The tuber of cassava will be cut up and dried up, milled or pelletized, and incorporated into the broiler’s diet and be capable of replacing 50% of the maize in their ration with no adverse effect on their performance; and the addition of 40% cassava flour or addition of 20% cassava peel meal in the layer’s ration was pleasing for laying performance [5].

The use of cassava tuber as an energy source has a certain weakness, namely the protein content of cassava tuber is lower than that of corn. Ngiki et al. [6] stated cassava tuber protein content was 1.3%, while the latest results showed a lower value of 1.14% [7]. Protein deficiency necessitates the addition of protein-rich feed ingredients. *Indigofera zollingeriana* leaf flour can be categorized as a protein source. According to Palupi et al. [8], *I. zollingeriana* shoot flour contains 28.98% crude protein, 8.49% crude fiber, and 3.30% crude fat. In addition to its high protein content, *I. zollingeriana* has many advantages, one of which is the presence of antioxidants because it contains carotenoids in the form of beta-carotene. Yadav et al. [9] reported cassava tubers could be used as an energy source to replace maize by 25% in starter phase broiler rations and 37.5% in finisher phase broiler rations.

Efforts can be made to maximize the efficiency of using alternative feed for livestock by adding an *acidifier* to increase digestibility. The type of *acidifier* that can be used as a feed additive is citric acid. Citric acid can lower the pH in the digestive tract, resulting in the formation of an acidic atmosphere and affecting the rate of digestion and the population of pathogenic bacteria in the digestive tract [10–14]. Deepa et al. [15] declared that the addition of 2% citric acid was able to increase feed consumption, increase body weight, and improve feed conversion in broilers, while other studies reported that lower citric acid supplementation of 1.5% had an impact on body weight, body weight gain, and carcass characteristics of broiler chickens [16].

In view of the depiction above, it is important to conduct research on the usage of cassava tubers and *I. zollingeriana* leaf (CIF) flour combination as an energy source to replace corn supplemented with citric acid in the ration on broiler production performance, carcass quality, and small intestine characteristics.

## Materials and Methods

### Animal ethics

An animal feeding experiment was conducted at the experimental station, Department of Animal Science, Faculty of Agriculture, Universitas Sriwijaya. The birds were cared for according to the Animal Welfare Guidelines of the Indonesian Institute of Sciences. The approval of the experiment was granted by Universitas Sriwijaya with approval number KPPHP-2021-1.

### Methods and sample preparation

This research used 100-day-old chick (DOC) broilers. The broilers were placed in 25 postal cages measuring 70 cm × 70 cm × 70 cm. Four broilers were placed in each cage. The cages had food dishes, water containers, and 60-watt incandescent lamps for lighting and warmth while the chicks are in them.

A grater, bucket, knife, plastic, rope, oven, tarpaulin, scales, measuring tape, and stationery were needed to make a cassava tuber and *I. zollingeriana* combination. A stereo microscope, pH meter, and viscocytometer were used in the laboratory observations. The feed ingredients used to prepare the ration in this study were CIF, concentrate, corn, fish flour, and rice bran. Subsequently, additional feed ingredients in the form of citric acid were added.

### Experimental design

This study used a completely randomized design with five treatments and five replications; each replication consisted of four broilers. The treatment was a substitution of corn in the ration with a combination of CIF: T0 (without CIF/control diet), T1 (5% CIF substitute for corn + 0.2% citric acid), T2 (10% CIF substitute for corn + 0.2% citric acid), T3 (15% CIF corn substitute + 0.2% citric acid), and T4 (CIF corn substitute + 0.2% citric acid).

### Making cassava tuber flour

The first step of making cassava tuber flour was to peel off the skin and wash the cassava. The cassava was then grated to aid in drying. Cassava was first dried in the sun and then in an oven at a temperature of 50°C for 24 h. The process of reducing the particle size of dried cassava was done by breaking the cassava into crumbles.

### Making I. zollingeriana leaf flour

To make *I. zollingeriana* leaf flour, *I. zollingeriana* leaves were harvested by separating the leaves from the twigs to dry them. Indigofera zollingeriana leaves were first dried in the sun and then in an oven at a temperature of 50°C until they were dry enough to be ground. The flour mill machine was used to process the dried I. zollingerian*a* leaves into flour.

### Ration

The feed ingredients used to prepare the treatment ration consisted of commercial feed in T0 treatment, concentrate, milled corn, fish flour, rice bran, a combination of cassava tubers, and *I. zollingeriana;* the composition had been arranged according to the treatment. After the treatment ration was mixed according to the composition of the ration, it was supplemented with 0.2% citric acid in the treatment using CIF. The nutritional composition of the ingredients of the ration is shown in Table 1, and the composition of the ingredients of the ration and the nutrient content of the research ration is shown in Table 2.

**Table 1.** Chemical composition.

Item	Chemical composition (%)							ME (cal/kg)
	CP	EE	CP	Ca	*P*	Lisin	Methionine	
Commercial feed	21.00	5.00	5.00	0.80	0.50	0.98	0.38	3,000
Rice bran	12.00	13.00	12.00	0.20	0.20	0.46	0.21	2,580.63
Corn	8.60	3.90	2.00	0.20	0.10	0.26	0.19	3,370.00
Concentrate	33.22	3.37	5.40	2.72	1.45	0.9	0.4	2,276.99
Fish flour	40.53	5.64	2.20	5.00	2.50	2.08	0.76	2,665.58
Cassava	01.10	0.55	2.30	0.32	0.71	0.08	0.04	3,519
*Indigofera Zollingeriana*	28.98	33.30	8.49	0.52	0.34	1.75	0.43	2,791.12
CIF	9.41	1.37	4.15	0.37	0.59	0.58	0.16	3,300.6

Ca, Calcium; CP, Crude Protein; CF, Crude Fiber; EE, Extract Ether; ME, Metabolism Energy; and P, Phosphor.

**Table 2.** Treatment ration composition.

Item	Treatments				
	T0	T1	T2	T3	T4
Commercial feed (%)	100	26	26.5	27.5	28
Rice bran (%)		40	35	30	25
Corn (%)		15	14.5	13.5	13
Concentrate (%)		14	14	14	14
Fish flour (%)		5	10	15	20
Total	100	100	100	100	100
Crude protein (%)	21.00	20.70	20.62	20.42	20.34
Extract ether (%)	5.00	6.40	6.32	6.29	6.21
Crude fiber (%)	5.00	5.17	5.31	5.48	5.62
Calcium (%)	0.80	1.19	1.21	1.21	1.22
Phosphor (%)	0.50	0.68	0.68	0.68	0.59
Lisin (%)	0.16	1.22	1.26	1.30	1.34
Methionine (%)	0.10	0.48	0.55	0.56	0.57
Metabolism energy (Ccal)	3,000	2,905.63	2,903.68	2,903.25	2,901.30

T0, without CIF; T1, corn substitution with 5% CIF; T2, corn substitution with 10% CIF; T3, corn substitution with 15% CIF; and T4, corn substitution with 20% CIF.

### Cage preparation

Before conducting the experiment, cages were cleaned of any dirt attached and limed evenly with the provided disinfectant. A disinfectant was sprayed evenly throughout the cage to eliminate germs and microorganisms that cause diseases. The cage was then left for 1 or 2 weeks. Clean feed and drinking stations, as well as other cage equipment, were placed in each cage. Each cage unit was labeled with treatment and replication.

### Rearing

Broiler DOC that had just arrived was given drinking water mixed with brown sugar with a concentration of 50 gm/l of water for the first 4 h as an energy source to restore its condition due to the stress of traveling from the hatchery to the rearing cage. Rearing was carried out for 4 weeks. The chicken ration was given in accordance with the treatment. Provision of feed and drinking water was done *ad-libitum* or continuously. Every day in the morning, the cage was cleaned. For DOC data collection, broilers were weighed once a week.

### Research data collection

Data on broiler chicken performance were collected once a week by measuring the weight gain of the chickens. Then the amount of feed given was weighed as well as the remainder of the feed.

### Observed variables

#### Feed Consumption (gm/head/days) = initial weight-final weight of ration (gm/cage)/amount of chicken (head/cage/days)

The ration consumption was calculated by weighing the ration given and the remaining ration every week. The following formula was used to calculate ration consumption per head per week [10].

#### Body weight gain (gm/head/days) = Final weight-Initial weight (gm/cage)/One week or Seven (days/cage/head)

Weekly body weight gain was measured by weighing the chickens at the end of the week. Weekly body weight gain can be calculated by using the following formula [10].

#### Feed Conversion ratio = (average feed consumption)/(average body weight gain) (FCR)

Feed conversion was calculated by dividing the average feed consumption in 1 week by the weekly average body weight gain. The ration conversion calculation was done by using the following formula [10].

#### Live weight

Live weight is obtained by measuring the weight of a chicken that has been fasted for 6 h to obtain an empty live weight before slaughtering [11].

#### Carcass percentage

Carcass percentage is the ratio of carcass weight to live weight multiplied by 100% [12].

#### Percentage of abdominal fat

The percentage of abdominal fat is calculated by multiplying the weight of abdominal fat by one hundred percent [13].

The number of small intestine villi was determined by the following method. Samples of the small intestine of the ileum which were 4–5 cm long were cut and the contents were removed. After that, the ileum was cleaned with NaCl solution and later stored in formalin solution with a concentration of 10%. Subsequently, the lumen of the small intestine was cut 4-µm thick using a microtome and placed on a slide for staining with the hematoxylin–eosin method. The samples were then observed under a microscope with a 40× magnification and the number of all villi (unit/transverse cut) was counted [14].

For measurement of digesta pH, 28-day-old broilers which were fasted for 6 h were used. The small intestine was separated from the ileum, and the contents of the small intestine were removed and put into a container for pH observation using a pH meter [15].

#### Digesta viscosity

After removing the digesta ileum, 1 gm of digesta was diluted with aquadest to a volume of 10 ml. The solution was centrifuged at 300 rpm for 5–10 min. A viscocytometer is used to measure the viscosity of the centrifuged supernatant liquid [16].

### Data analysis

The data obtained were processed using Statistical Product and Service Solutions software version 20 based on the design used. In case there were differences between treatments, the Duncan new multiple range test was tested.

## Results and Discussion

Table 3 shows the findings of the experiment on the effects of feeding broilers a mixture of CIF flour supplemented with citric acid in the ration.

**Table 3.** Effect of feed treatment on performance, carcass and quality of the digestive tract.

Variables	Treatments				
	T0	T1	T2	T3	T4
Consumption (gm/head/day)	54.91 ± 2.29	56.86 ± 2.22	56.99 ± 1.33	55.17 ± 1.69	54.96 ± 1.61
Body weight gain (gm/head/day)	42.44^a^ ± 3.42	43.91^a^ ± 2.35	41.61^a^ ± 3.03	37.36^b^ ± 2.60	36.60^b^ ± 2.63
FCR	1.301^a^ ± 0.122	1.297^a^ ± 0.074	1.375^a^ ± 0.112	1.480^b^ ± 0.070	1.508^b^ ± 0.119
Live weight (gm)	1368^a^ ± 98.40	1,316.8^ab^ ± 49.39	1,227.8^ab^ ± 146.66	1,175.2^bc^ ± 123.45	1,153.6^c^ ± 226.64
Carcass (%)	72.36^a^ ± 2.37	71.57^ab^ ± 1.50	70.67^ab^ ± 0.73	67.72^c^ ± 4.29	68.90^c^ ± 1.90
Abdominal fat (%)	1.22 ± 0.36	1.16 ± 0.46	0.76 ± 0.27	1.16 ± 0.17	1.29 ± 0.30
Number of villi	244.4^a^ ± 10.96	286.4^b^ ± 10.21	273.0^b^ ± 11.29	240.6^a^ ± 8.87	241.8^a^ ± 2.86
pH digest	5.56 ± 0.15	5.56 ± 0.32	5.80 ± 0.14	5.66 ± 0.11	5.86 ± 0.11
Viscosity	1.93 ± 0.42	1.95 ± 0.52	1.91 ± 0.35	2.29 ± 0.42	2.15 ± 0.20

FCR, Feed conversion ratio; T0, without CIF; T1, corn substitution with 5% CIF; T2, corn substitution with 10% CIF; T3, corn substitution with 15% CIF; T4, corn substitution with 20% CIF.

Means with different superscript letters in the same line differ significantly (*p* < 0.05).

### Feed consumption

According to the results of the analysis of variance, adding citric acid to the flour made from CIF did not significantly affect ration consumption (*p* > 0.05). In this study, the average daily ration consumption ranged from 54.91 to 56.99 gm/head/day. Feed consumption in the study was higher when compared to the research results of Chang’a et al. [17], who reported that the average consumption of cassava flour feed with the addition of the *Ronozyme* enzyme in broiler feed was 44.94–46.77 gm/head/day. The treatment outcomes, which had no significant influence on ration consumption, were consistent with the study’s findings by Yadav et al. [9], who stated that the consumption of a ration using cassava tubers as a substitute for corn at a level of 50%, which had a metabolic energy content of 2,878 kcal/kg and a protein content of 20.61%, had the same effect as the control ration. Another study also reported the substitution of 50% cornstarch in broiler rations without adverse effects on chicken appearance, and the expansion of 40% cassava flour or the expansion of 20% cassava peel flour in laying hen rations could improve the performance of laying hens [5].

This proved that the combination of CIF flour had high palatability and nutritional quality, allowing broilers to respond well to treatment rations. Treatments that had no significant effect on ration consumption revealed that using CIF flour combinations at 5%, 10%, 15%, and 20% did not cause physical, taste, or odor differences, and hence, broilers liked them and did not cause a decrease in palatability. According to Akhadiarto [18], the palatability of the feed, which is regulated by the smell, taste, and form of the components that make up the ration, affects how much feed is consumed. Furthermore, the nutritional quality of all ration treatments was the same, ensuring that the protein and energy balance consumed by broilers was adequate. This is consistent with the statement of Ahmed and Arabi[19], which states that giving rations with relatively the same energy content will have the same effect on ration consumption.

Hydrogen cyanide (HCN) is an antinutritional compound found in cassava tuber feed components, whereas tannins and saponins are antinutritional compounds found in *I. zollingeriana* leaf flour. These antinutritional substances have no effect on ration consumption when a combination of CIF flour is used up to 20% in broiler rations during rearing. According to Jayanegara et al. [20], tannins and saponins contain antinutrients with an astringent taste. The astringent taste of antinutrients in treatment rations was tolerated by broilers and did not reduce the palatability of the treated rations.

### Body weight gain

According to the results of the analysis of variance, adding citric acid to a ration that included CIF flour had a significant effect (*p* < 0.05) on broiler body weight gain. In this study, the average body weight gain of broiler chickens was 36.60–43.91 gm/head/day. Body weight gain in this experiment was slightly higher than that described by Hossain et al. [21], who reported an average of 40.44–41.07 gm/head/day with cassava provision in broiler rations. In accordance with the findings of Ojewola et al. [22], using 10% cassava tubers as a substitute for corn has the same effect on body weight gain in broilers, but giving 20%–100% cassava tubers could reduce body weight gain. However, the results of other studies reveal that the use of cassava tuber can be increased by up to 50%–75% if the fermentation process is followed [2].

The use of CIF flour combination of 5%–10% as a substitute for corn had no significant impact (*p* > 0.05) on body weight gain, despite the fact that both T1 and T2 contained antinutritional substances. T1 contained tannins up to 0.04 gm/kg, saponins of 0.00054 mg/kg, and 0.14 mg/kg HCN. Meanwhile, T2 contained 0.08 gm/kg tannins, 0.0010 mg/kg saponins, and 0.28 mg/kg HCN. Even though there were antinutritional substances in T1 and T2, 0.2% citric acid supplementation improved broiler digestion and had the same effect on broiler body weight gain as the control treatment. *Acidifier* serves to accelerate the acidification status of the digestive tract so that protein-digesting enzymes can work more quickly and become active [12–14]. The use of CIF 15%–20% flour combination as a substitute for corn had a significant impact (*p* < 0.05) on body weight gain in broilers.

An increase in the content of antinutritional substances, specifically tannins, saponins, and HCN at T3 and T4, was the cause of the decreased body weight gain in broiler chickens. *Indigofera zollingeriana* leaf flour contributed 0.13–0.17 gm/kg tannins, 0.0016–0.0021 mg/kg saponins, and 0.42–0.56 mg/kg HCN at T3 and T4. Tannins can bind to proteins, reducing protein digestibility. However, the complex bonds of tannins with proteins can be released at low pH in the digestive tract, allowing the protein to be degraded by digestive enzymes and the amino acid content to be utilized by livestock [23,24]. Furthermore, saponins are considered to have an inhibitory effect on livestock growth because they inhibit the activity of a number of gastrointestinal enzymes such as trypsin and chymotrypsin [25]. Additionally, the HCN in the ration can inhibit the production of ATP, resulting in a lack of energy in livestock [20]. Citric acid supplementation with a dose of 0.2% at a 15%–20% substitution using corn has also not been able to work properly due to an increase in antinutritional substances, resulting in less than ideal nutrition absorption in the digestive system.

### Ration conversion

The findings of the analysis of variance showed that adding citric acid to a ration that contained CIF flour had a significant impact (*p* < 0.05) on the ration’s conversion value. In this investigation, the average ration conversion value was 1.29–1.5. The results of the ration conversion in this experiment were less encouraging than those reported by Rahmadani et al. [26], who reported that the ration conversion value had an average of 1.88–1.96 with the addition of cassava with *isoamylase* to broiler rations. In addition, Yadav et al. [9] stated that the use of cassava tuber as a substitute for corn at 50% in broiler rations had an average conversion rate of 1.59–1.82. This is in line with what Ojewola et al. [22] found, who argued that broilers fed with cassava tuber rations at a 5% level as a substitute for corn have no negative effect on the conversion value of the ration.

According to the results of a study, replacing corn up to 10% with a combination of CIF had the same effect on broiler ration conversion. This is because T1 and T2 had the same impact on body weight gain and ration consumption. In addition, 0.2% citric acid supplementation was able to provide a maximum effect on the digestive tract, so the use of cassava tuber up to 10% could be digested well by broilers. The reason was that both T1 and T2 had a similar impact on body weight gain and ration consumption. Moreover, 0.2% citric acid supplementation was able to provide the best effect on the digestive tract, allowing broiler chickens to digest cassava tubers up to 10%. Citric acid supplementation in the ration ranged from 0.25%–1% increased feed conversion in broilers [27,28].

The high value of ration conversion in T3 and T4 treatments was due to the low body weight gain of broilers despite consuming the same amount of ration. Furthermore, 0.2% citric acid supplementation did not improve nutrient digestibility in the T3 and T4 treatment rations due to an increase in antinutritional substances. According to Allama et al. [29], a low ration conversion value means that the feed efficiency of the broilers is good since the broilers use feed to produce meat more effectively.

### Live weight

The findings of the analysis of variance showed that using a combination of cassava tuber and *I. zollingeriana* leaf flour supplemented with citric acid in the ration had significant results (*p* < 0.05) on live weight. The average live weight of broiler chickens in this study was 1153.6–1368 gm/head. In this investigation, the live weight value was higher than the findings of Chang’a et al. [17], in which the average live weight of 24-day-old broilers fed with a 25%–75% substitute for corn with cassava tuber flour as the main source of energy was 1171.3–1354.7 gm/head. But this value is less than what Uguru et al. [2] found when they fermented cassava tubers.

Starch makes up the majority of the nutrients found in cassava tubers. In terms of carbohydrates, amylose and amylopectin make up the glucose polymer known as starch [30]. The high content of amylopectin in cassava tubers causes the starch content to be easily digested by broilers, even better than corn. The use of a combination of CIF flour as much as 20% of the ration will cause an increase in antinutrients that decreases live weight. The live weight of a broiler is influenced by the ability of the broiler to convert the ration into meat, which is hampered by the presence of cyanide in the ration. Obviously, this is not the best way to absorb nutrients, so the nutrients that are taken in are not turned into meat in the best way, which affects the live weight of broilers [31].

The use of *I. zollingeriana* leaf powder at T1 and T2 contributed 0.14–0.28 mg/kg of HCN. T3 and T4 contributed 0.42–0.56 mg/kg of HCN. The content of antinutrients such as cyanide acid (HCN) in cassava tubers is around 17.5 mg/kg. Processing of cassava is necessary to avoid cyanide poisoning [32]. Linamarin, also known as phaseolunatin, is an illustration of a cyanogenetic glycoside and can be found in linseed, Java beans, and cassava. Because HCN is toxic and is released by the hydrolysis of cyanogenetic glycosides, plants that contain these glycosides may be harmful to animals. Before poisoning sets in, the glycoside must be hydrolyzed because it is not dangerous by itself. A commonly found enzyme in plants, on the other hand, may easily degrade the glycoside to its constituent parts [33]. The addition of 20% CIF flour supplemented with 0.2% citric acid did not increase broiler live weight. This is because adding 0.2% citric acid to the treatment ration could not handle the lack of nutrients.

### Carcass percentage

The application of CIF flour combined with citric acid in the ration produced significant results (*p* > 0.05) on the percentage value of broiler carcasses, according to the results of the analysis of variance. The average proportion of broiler carcasses in this investigation ranged from 68.90% to 72.3%. Khempaka et al. [34] reported that the average percentage of broiler carcasses aged 28 days fed with 4%–20% fermented cassava tuber feed *Aspergillus oryzae* was 66.10%–67.42%. While the results of other studies showed that fermented cassava tubers given between 50%–75% of the ration for 56 days had a significant influence on the average value of carcass percentage[2].

The content of metabolic energy in the ration is expected to stimulate broiler growth, resulting in a high live weight and a high carcass weight. According to a study by Solikin et al. [35], carcass weight is directly correlated with the final weight of the chicken; the higher the chicken’s weight, the higher the carcass weight. However, the percentage of carcass decreased with the use of a combination of CIF flour with different compositions for each treatment ration even though the metabolic energy of all rations were kept the same. Nevertheless, due to its low protein content, imbalanced amino acid profile, high fiber content, and presence of antinutrients, particularly cyanogenic glucosides, the use of cassava tubers in chicken feed are restricted (HCN). Additionally, there are other antinutrients such as tannins and saponins that have been discovered in *I. zollingeriana* leaf flour. The use of *I. zollingeriana* leaf flour in treatments T1 and T2 contributed 0.04–0.08 gm/kg tannins and 0.00054–0.0010 mg/kg saponins. Meanwhile, the use of *I. zollingeriana* leaf flour in treatments T3 and T4 contributed 0.13–0.17 gm/kg tannins and 0.0016–0.0021 mg/kg saponins. Furthermore, Palupi et al. [8] mentioned that there are 0.29% tannins and 0.036 ppm saponins *in I. zollingeriana* shoot flour. The chemical nature and dosage of tannins determine their antinutritional effects. Tannins are heat stable, and they reduce the ability of both animals and people to digest protein. This was probably due to the fact that they either made protein partially inaccessible or inhibited digestive enzymes, which increased fecal nitrogen. The bitterness and throat-irritating properties of saponins, on the other hand, led to their recognition as antinutrient elements because of their negative impacts, including growth retardation and reduced food intake. Furthermore, saponins have been found to reduce supplement bioavailability and enzymatic activity, and it influences protein absorbability by inhibiting stomach-related enzymes such as trypsin and chymotrypsin [25].

As a result, the presence of citric acid in the treatment ration aided in the absorption of *I. zollingeriana* leaves. Citric acid accelerates the acidification of the digestive tract, allowing protein-digesting enzymes to work more quickly and actively [36,37]. The feeding of 15%–20% CIF flour supplemented with 0.2% citric acid did not increase the percentage of broiler carcasses. This was because adding 0.2% citric acid to the treatment ration did not make it easier for the animal to absorb nutrients from the food.

### Abdominal fat percentage

The analysis of variance showed that using CIF flour combination supplemented with citric acid in the ration showed no significant impact (*p* > 0.05) on the percentage of abdominal fat in broiler chickens. The average percentage of broiler abdominal fat in this study was 0.76%–1.29%. Compared to Khempaka et al. [34], this study’s percentage value of abdominal fat was higher, which means the average percentage of abdominal fat of 28-day-old broiler chickens fed fermented cassava tuber *A. oryzae* with 4%–20% feeding is 0.73%–1.09%. Cabel and Waldroup [38] reported that the percentage of abdominal fat in broiler carcasses is normally between 0.73% and 3.78%. This shows that the treatment of cassava tubers could not reduce abdominal fat deposits because the energy content of each ration was the same, so the rate of energy accumulation in the broiler body in the form of body fat was the same between treatments. Subekti et al. [39] stated that the formation of abdominal fat in the bodies of chickens occurs due to excess energy obtained from the feed they consume. The high crude fat content of the diet might also lead to the development of abdominal fat. The nutritional requirements of broiler chickens must contain 3%–4% crude fat in the ration, while in the combination of CIF flour in the crude fat treatment ration it is 5%–6%. Fouad and El-Senousey [40] also mentioned the relationship between abdominal fat and total carcass fat; that is, the higher the abdominal fat concentration, the higher the carcass fat content. Fat in the body of a chicken is derived from feed and is produced through the process of fat synthesis in the feed.

According to the findings of this study, the combination of CIF flour supplemented with 0.2% citric acid in the treatment ration on the broilers’ abdominal fat was given 5%–20%, but did not result in excess metabolic energy in the ration. Adding 0.2% citric acid to the treatment ration increased the abdominal fat percentage. This is in contrast to the research of Fik et al. [16], which found that 0.5%–1.5% citric acid in feed can reduce the rate of broiler abdominal fat because of the differences in ration energy content and dosage in broiler rations.

### Small intestine villi number

According to the analysis of variance results, adding citric acid to the diet along with CIF flour had a significant impact (*p* < 0.05) on the number of ileum villi in broilers. The average number of ileum villi in broiler chickens in treatment T0 was significantly different from treatments T1 and T2 (*p* < 0.05), according to the outcomes of additional tests. This is because for the T1 and T2 treatments, 0.2% citric acid supplementation was added, although the pH of the ileum digesta obtained was the same as the T0 treatment (control). Nevertheless, adding citric acid effectively increased the number of small intestinal ileum villi in broilers. Hidayat et al. [41] stated that *acidifier* (citric acid) in general could replace the role of antibiotics and were effective in increasing the absorption of food extracts in the small intestine.

The results of further tests on the T0 treatment were not significantly different (*p* > 0.05) from the T3 and T4 treatments. This is because, in the T3 and T4 treatments, there was an increase in the amount of CIF (a combination of CIF flour) by 15%–20%, which means an increase in the concentration of antinutrients in the ration, namely HCN (cyanic acid), tannins, and saponins. Even though the T3 and T4 treatments contained 0.2% citric acid supplementation, the supplemented citric acid did not increase the number of villi as in the T1 and T2 treatments.

Treatments at T1 and T2 had different effects (*p* < 0.05) with treatments T3 and T4. This was due to the increasing concentration of antinutrients at T3 and T4. However, the concentration of antinutrients (HCN, tannins, and saponins) in this study was still within the limits that broilers could tolerate. Jayanegara et al. [20] stated that the maximum limit of HCN content in poultry is 10 mg HCN/kg feed. Still, a low concentration of antinutrients could affect the number of villi in this study’s ileum of the small intestine of broilers. The presence of antinutrients in the ration caused the number of small intestinal villi to be lower when compared to the research of Kusuma et al. [42], which reported that the lowest number of intestinal villi in the research of alternative feeds using a total of 60% palm kernel cake and onggok fermented food in the ration was 386.60 ± 19.91 μm.

### Digesta pH

The variance analysis showed that using a combination of CIF flour supplemented with citric acid in the ration showed no significant impact (*p* > 0.05) on the pH value. The average pH value in this study ranged from around 5.56 to 5.86. The partial replacement of corn as an energy source in the ration with a combination of cassava tubers and *I. zollingeriana* did not affect the pH value of the small intestine digesta until the feeding of a 20% combination of cassava tubers and *I. zollingeriana* in the ration. However, the pH value results in the ileum were relatively low, at 5.56–5.86, when compared to the ileum, which generally had a high pH value, which was at 6–7. Based on research by Mabelebele et al. [43], it is reported that ideally, the pH value in broiler chickens ranges from 3.47 in the gizzard to 6.43 in the small intestine.

Organic acid has a role in increasing the activity of proteolytic enzymes in the digestive tract and can also increase the natural immune response in poultry [27,37,44]. The pH value and viscosity value are essential factors influencing the flow rate of nutrients in the digestive tract. This causes the digestion rate to be fast and allows a decrease in the digestive process. The absorption of nutrients becomes less effective, reducing the availability of nutrients for the synthesis of body tissues. According to Cahyaningsih et al. [45], a low degree of acidity in the digestive system (stomach and intestines) can optimize the absorption of nutrients in the stomach and intestines because it can slow down the rate of digestion, resulting in the optimization of feed nutrient absorption. It does not interfere with the digestive process and nutrient utilization, so the growth of harmful bacteria is inhibited [37,44,46].

### Viscosity

The outcome of the analysis of variance showed that the combination of CIF flour supplemented with citric acid in the diet showed no significant impact (*p* > 0.05) on digesta viscosity. The average digesta viscosity in this experiment was from (1.91 d.Pas ± 0.35) to (2.29 d.Pas ± 0.42). The viscosity in this study was higher than that of Emma et al. [47], where the lowest viscosity value was (0.10 ± 0.004 d.Pas), and the highest viscosity was (0.23 ± 0.008 d.Pas).

The viscosity value in the digestive tract is directly proportional to the high or low pH value. Negative effects will appear if the viscosity of the small intestine increases, which will then reduce digestion efficiency by slowing the diffusion rate of endogenous enzymes to react with nutrients, as well as improve blood biochemistry in quail and broilers [48]. The level of viscosity influences the value of viscosity. The higher the viscosity level, the worse the viscosity value will be. Inversely, the viscosity value is adequate if the viscosity level is low or watery [49].

Viscosity significantly impacts the value of performance data for poultry, with high or low viscosity values affecting the value of feed digestibility and feed flow rate in the digestive tract. Furthermore, changes in the viscosity value can occur due to the type of feed consumed, such as feed with a high solubility value, which can result in a low viscosity value in treatment. This occurrence is thought to be caused by the influence of *non-starch polysaccharides* (NSP) solubility value in feed ingredients. According to Saputra et al. [50], NSP, resistant starch, and short-chain carbs are the most common forms of carbohydrates that pass through the colon without being hydrolyzed.

## Conclusion

In place of corn in the ration, a 10% mixture of CIF flour can be used as an energy source without affecting the broilers‘ consumption of the ration, body weight gain, ration conversion, live weight, percentage of the carcass, percentage of abdominal fat, digesta pH, viscosity, or several villi in the small intestine.

## List of Abbreviations

CIF, cassava tuber and *Indigofera zollingeriana* leaf; CRD, completely randomized design; DOC, day-old chick; C, control (basal diet); T0, without CIF/Control Diet; T1, 5% CIF substitute for corn + 0.2% citric acid; T2, 10% CIF substitute for corn + 0.2% citric acid; T3, 15% CIF corn substitute + 0.2% citric acid; T4, CIF corn substitute + 0.2% citric acid; FCR, feed conversion ratio.
